# Resource Availability as Driving Factor of the Reproductive Mode in Soil Microarthropods (Acari, Oribatida)

**DOI:** 10.1371/journal.pone.0104243

**Published:** 2014-08-06

**Authors:** Katja Wehner, Stefan Scheu, Mark Maraun

**Affiliations:** 1 Institute of Zoology, University of Technology, Darmstadt, Germany; 2 Institute of Zoology und Anthropology, Georg August University, Göttingen, Germany; Roehampton university, United Kingdom

## Abstract

The availability of high quality resources is an important factor driving community structure and reproductive mode of animals. Parthenogenetic reproduction prevails when resources are available in excess, whereas sexuality correlates with resource shortage. We investigated the effect of resource availability on the community structure of oribatid mites in a laboratory experiment. Availability of food resources was increased by addition of glucose to leaf litter and reduced by leaching of nutrients from leaf litter. Experimental systems were incubated at three different temperatures to establish different regimes of resource exploitation. Community structure of oribatids and numbers of eggs per female were measured over a period of ten months. We expected the density of oribatid mites to decline in the reduced litter quality treatment but to increase in the glucose treatment. Both effects were assumed to be more pronounced at higher temperatures. We hypothesized sexual species to be less affected than parthenogenetic species by reduced resource quality due to higher genetic diversity allowing more efficient exploitation of limited resources, but to be outnumbered by parthenogenetic species in case of resource addition due to faster reproduction. In contrast to our hypotheses, both sexual and parthenogenetic oribatid mite species responded similarly with their densities declining uniformly during incubation. The parthenogenetic Brachychthoniidae and *Tectocepheus* dominated early in the experiment but were replaced later by parthenogenetic Desmonomata and *Rhysotritia*. In parthenogenetic species the number of eggs per female increased during the experiment while the number of eggs in sexual females remained constant or decreased slightly; in general, egg numbers were higher in sexual than in parthenogenetic species. The results indicate that for sustaining oribatid mite populations other resources than litter and associated saprotrophic microorganisms are needed. They also indicate that there are two groups of parthenogenetically reproducing species: exploiters of easily available resources and consumers of leaf litter associated resources.

## Introduction

The amount and availability of resources is one of the most important factors structuring animal communities. In addition to changing population density and community structure it also influences the reproductive mode of animals. Parthenogenesis dominates when resources are available in excess whereas sexuality correlates with resource shortage [1], [2]. Theoretical models explaining the prevalence of sex in the animal kingdom, such as the Tangled Bank [3] or Lottery Models [4], imply that the response of individuals with different genotypes to environmental heterogeneity differs. Sexual species are likely to occupy a wider range of environmental conditions and to exploit a wider range of resources as they include diverse genotypes due to outcrossing and recombination [1]. Consequently, theory predicts the dominance of sexuality in unstable habitats with varying environmental conditions [4]–[6]. On the other hand, parthenogenesis should be favoured in stable habitats (such as the litter layer or deep soil) with constant availability of resources [2]. Fluctuations in resource quantity and quality therefore are likely to influence the predominance of sexual or parthenogenetic taxa [7], [8]; that sexuality in fact often is associated with resource shortage is exemplified by cyclical parthenogenesis such as in aphids and cladocerans switching to sexual reproduction when resources diminish by the end of the season [1], [9].

Recent theoretical considerations suggest that sex prevails when resources are difficult to obtain and in short supply, implying that exploited resources will not be available to the same extent and same quality to the consumer in the next generation [2], [10]. Based on these considerations parthenogenesis is favoured when death rates are high, resources are homogeneous, resources regenerate fast or several genetically diverse parthenogenetic species cover the entire spectrum of resources. Accordingly, the higher percentage of parthenogenetic species in forest soils as compared to other terrestrial ecosystems is likely due to ample availability of resources with resources being “regenerated” in pulses via litter material [11]–[13] and root associated materials such as exudates and mycorrhizal fungi [14], [15]. The variety of food resources available, including leaf and root litter, bacteria, fungi, algae and lichens, allows the coexistence of a large number of soil meso- and macrofauna species [16], [17]. However, it is puzzling why in many soil taxa sexually and parthenogenetically reproducing species of close taxonomic affiliation and similar trophic niche coexist in the same habitat [18]–[20].

An important soil-dwelling group of microarthropods are oribatid mites (Oribatida, Acari). They play a key role in decomposition of litter and consume a variety of food resources [17]. A remarkable feature of this speciose group of soil invertebrates is the high incidence of parthenogenesis; about 10% of all oribatid mite species have abandoned sex [21] and in forest soils of the temperate and boreal zone typically 60–80% of the individuals reproduce by parthenogenesis [21]–[25]. Based on the high percentage of parthenogenesis and the high age [26]–[29], some taxa of oribatid mites join bdelloid rotifers [30] and darwinulid ostracods [31] as “ancient asexual scandals” [32], [33], challenging evolutionary theories that predict the extinction of parthenogenetic species in the long-term. The ubiquity of oribatid mites, their high abundance in forest soils and the co-existence of sexual and parthenogenetic species in the same habitat coupled with a complexity of K- and r-selected life cycles [21], [34]–[36], presents oribatid mites as ideal model group for investigating evolutionary questions, such as the advantage of sexual reproduction.

One possible explanation for the coexistence of sexual and parthenogenetic oribatid mite species in forests is that they colonize different microhabitats. Indeed, oribatid mite species on the bark of trees are mainly sexual [37], whereas parthenogenetic species predominantly live in soil [19], [24], [25]. This correlates with the assumption that sex prevails in unstable habitats (the bark of trees) [38] while parthenogenesis is favoured when conditions are stable as it is the case in soil; recent studies on oribatid mites have shown that parthenogenetic taxa indeed suffer more from resource limitation than sexual species [25].

The present study investigates the influence of changes in the availability of resources on oribatid mite density, community structure and reproductive mode at different temperatures in a laboratory microcosm experiment. Therefore, treatments with untreated litter material, with litter of reduced quality and with glucose-enriched high quality litter material were established. Microcosms were incubated at different temperatures as we expected effects to be more pronounced at higher temperatures due to faster exploitation of resources and accelerated reproduction. In general, we expected the number of specimens to decline in the reduced litter quality treatment due to declining resources but to increase in the glucose enriched treatment due to increased food availability, in particular microorganisms and resources mobilized by them. We hypothesized that parthenogenetically reproducing species will be favoured in the high quality litter treatment due to faster reproduction caused by the avoidance of searching mating partners and investing in the production of males. Consequently, when resources become limiting we expected sexuals to become more dominant at the expense of parthenogenetic species since sexual populations presumably comprise more diverse genotypes allowing more complete resource exploitation.

## Materials and Methods

### Study site

Soil samples were taken from the Kranichstein forest, located about 8 km northeast of Darmstadt (Germany; 49°53′40.2″N 8°42′23.1″E). The Kranichstein forest is dominated by beech (*Fagus sylvatica*) interspersed with ca. 190 y old oak (*Quercus robur*) and hornbeam (*Carpinus betulus*). The herb layer is dominated by *Luzula luzuloides, Milium effusum, Anemone nemorosa* and *Polytrichum formosum*. Parent rock is Rotliegendes covered with sand; the humus form is moder (FAO-UNESCO classification).

For taking the samples, no specific permission was required but prior to sampling the forestry agency of Darmstadt (Forstamt Darmstadt) was informed and agreed. The study did not involve endangered or protected species.

### Experimental setup

Soil cores (45 in total; Ø 21 cm L, F, H layer and the upper 3 cm of the Ah layer) were taken from the Kranichstein forest and placed in laboratory microcosms. The litter material of all soil cores was removed, mixed carefully and weighted. Subsequently, equivalent amounts of litter were placed back into the microcosms establishing three treatments with five replicates each: (a) control treatment with untreated litter material, (b) reduced litter quality treatment with litter kept in water at a temperature of 60°C for 24 h to reduce food quality by leaching of easily available carbon compounds, and (c) high quality litter treatment with untreated litter sprinkled with glucose solution as additional carbon source for microorganisms at regular intervals. We expected additional glucose to be beneficial for microorganisms and thereby also for oribatid mites feeding on detritus and associated microorganisms. As baseline first samples for analyzing oribatid mite communities were taken short after the first addition of glucose. Microcosms were incubated at constant 10, 15 and 20°C in darkness. Microcosms were closed with plastic foil at the bottom and with gauze (45 µm) at the top; loss of water was evaluated gravimetrically and replaced by distilled water every week for the control and reduced litter quality treatment or by adding glucose solution for the glucose enriched treatment.

As a measure of the resource status of the microcosms, microbial respiration was measured as CO_2_ produced and mineralization of carbon was calculated as mg C microcosm^−1^ week^−1^. CO_2_ evolved in the microcosms was trapped in 2 ml alkali (1 M NaOH) in vessels placed on the leaf litter. Trapped CO_2_ was measured in an aliquot by titration with 0.1 M HCl after precipitation of carbonate with saturated BaCl_2_ [39]. Microbial C mineralization was used to calculate the amount of carbon produced and subsequently replenished by adding the 2-fold amount of glucose to the glucose treatment.

After 2, 10, 20 and 44 weeks soil cores (Ø 5 cm) were taken from the microcosms, separated into litter and soil (0–3 cm depth) and oribatid mites were extracted by heat [40], [41]; holes in soil were filled with sand. Oribatid mites were counted and determined to species level if possible. In addition, adults were sexed, and in females the number of eggs was counted to compare egg production between parthenogenetic and sexual species. Species were considered to be sexual when there were more than 5% males. Brachychthoniidae, Phthiracaridae and Suctobelbidae were difficult to sex; females could only be identified if they carried eggs. The mode of reproduction in these taxa was inferred from the literature [24].

### Aggregation of species

Species of adult oribatid mites were aggregated into ten subgroups according to phylogenetic relationships and the inferred mode of reproduction: (1) Brachychthoniidae (parthenogenetic, including undetermined Brachychthoniidae, *Brachychthonius berlesei*, *Liochthonius* sp. and *Sellnickochthonius honestus*), (2) Enarthronota (parthenogenetic, comprising only *Eniochthonius minutissimus*; excluding Brachychthoniidae), (3) Phthiracaridae (undetermined species, mainly sexual, except *Rhysotritia duplicata*), (4) *Rhysotritia duplicata* (parthenogenetic), (5) Desmonomata (parthenogenetic, comprising *Malaconothrus gracilis, Nanhermannia coronata, N. nana* and *Nothrus silvestris*), (6) *Tectocepheus* (parthenogenetic, comprising *T. minor, T. sarekensis* and *T. velatus*), (7) Suctobelbidae (parthenogenetic, including undetermined Suctobelbidae, *Suctobelbella subcornigera* and *S. subtrigona*), (8) parthenogenetic Oppiidae (including *Oppiella nova* and *Microppia minus*), (9) sexual Oppiidae (including *Berniniella sigma, Disshorina ornata* and *Medioppia subpectinata*) and (10) other Circumdehiscentiae (sexual, including *Achipteria coleoptrata, Cultroribula bicultrata, Carabodes femoralis, C. ornatus, Galumna lanceata*, *Ophidiotrichus tectus, Oribatula tibialis, Minunthozetes semirufus* and *Banksinoma lanceata*). For full species names see Table S1.

### Statistical analysis

For statistical analyses specimens of the litter and soil layer were combined; the great majority of individuals occurred in the litter layer. Changes in time of microbial C mineralization, density of total oribatid mites, density of subgroups of oribatid mites, percentages of parthenogenetic individuals, and numbers of eggs per female were analysed by repeated measures multivariate analysis of variance (RM-ANOVA) or repeated measures general linear models (RM-GLM) in SAS 9.1.3 (SAS Institute Inc., Cary, USA), with time as repeated and treatment and temperature as categorical factors [42]; changes in density of oribatid mite subgroups were previously analysed for the interaction between time, taxa, treatment and temperature by a double repeated ANOVA with the repeated factors “taxa” and “time”.

Further, changes in oribatid mite community composition were analysed by non-metric multidimensional scaling (NMDS) [43] implemented in Statistica (StatSoft Inc., Tulsa, USA). NMDS reduced the number of meaningful dimensions to three which were used as independent factors in a subsequent discriminant function analysis (DFA) to explore effects of time. In addition, data on oribatid mite species were analysed by detrended correspondence analysis (DCA) using CANOCO 4.5 [44].

Abundances of individuals, numbers of eggs per female and data on microbial C mineralization were log(x+1) transformed and percentages of parthenogenetic individuals were arcsin square root transformed prior to statistical analysis to increase homogeneity of variances. Species with fewer than ten individuals in total and species which occurred in less than three samples were excluded from the statistical analyses (Table S1). All data are available upon request from the corresponding author.

## Results

### Microbial C mineralization

**Figure 1:**
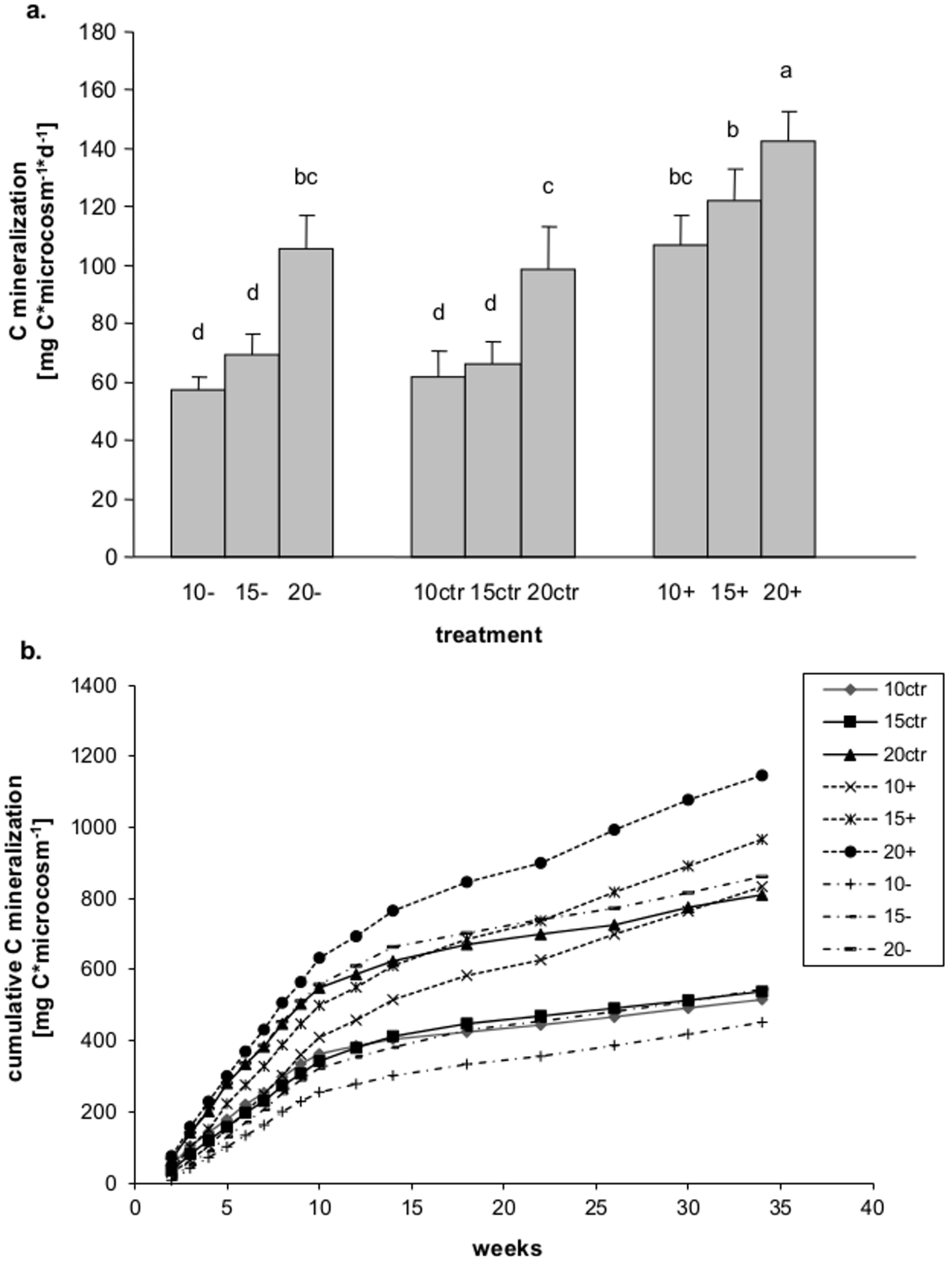
Carbon mineralization. (a) Average daily carbon mineralization as affected by resource manipulation (control, reduced litter quality, glucose addition) and temperature (10, 15, 20°C), and (b) cumulative carbon mineralization during 34 weeks of incubation in the respective treatments. “ctr” =  control (solid line), “−” =  reduced litter quality (broken line), “+” =  glucose addition (dashed line).

Average daily microbial C mineralization was at a maximum at 20°C (ANOVA, F_2,36_ = 141.6, P<0.0001 for temperature) and increased after glucose addition (ANOVA, F_2,36_ = 101.5, P<0.0001 for treatment; Fig. 1a). Cumulative microbial C mineralization per microcosm increased linearly from week 2 to 10 and then continued at lower rates until week 35 (Fig. 1b). This reduction after 12 weeks was less pronounced in the glucose treatment whereas respiration rates in the control and reduced litter quality treatment were similar (RM-ANOVA, F_60,540_ = 2.35, P<0.0001 for the interaction between time, temperature and treatment).

### Density and community composition of oribatid mites

In total, 25,639 oribatid mites were inspected; 12,224 individuals were juvenile and not determined further. The 13,415 adult oribatid mites represented 33 species and three pooled taxa (undetermined Brachychthoniidae, Phthiracaridae and Suctobelbidae); of these, 13,401 individuals from 28 species and the three pooled taxa were included in the statistical analysis (Table S1).

Among the 28 species included in the analysis 12 were sexual with percentages of females ranging from 29% in *Carabodes ornatus* to 90% in *Cultroribula bicultrata* and 16 were parthenogenetic comprising 100% females except in *Oppiella nova* (98%) and *Tectocepheus minor* (99%; Table S1). The percentage of total parthenogenetic individuals neither changed with time (RM-GLM, F_3,81_ = 1.3, P = 0.266) nor differed between treatments (RM-GLM, F_2,27_ = 2.9, P = 0.074) nor was affected by temperature (RM-GLM, F_2,27_ = 1.2, P = 0.311); it was on average 84%.

**Figure 2:**
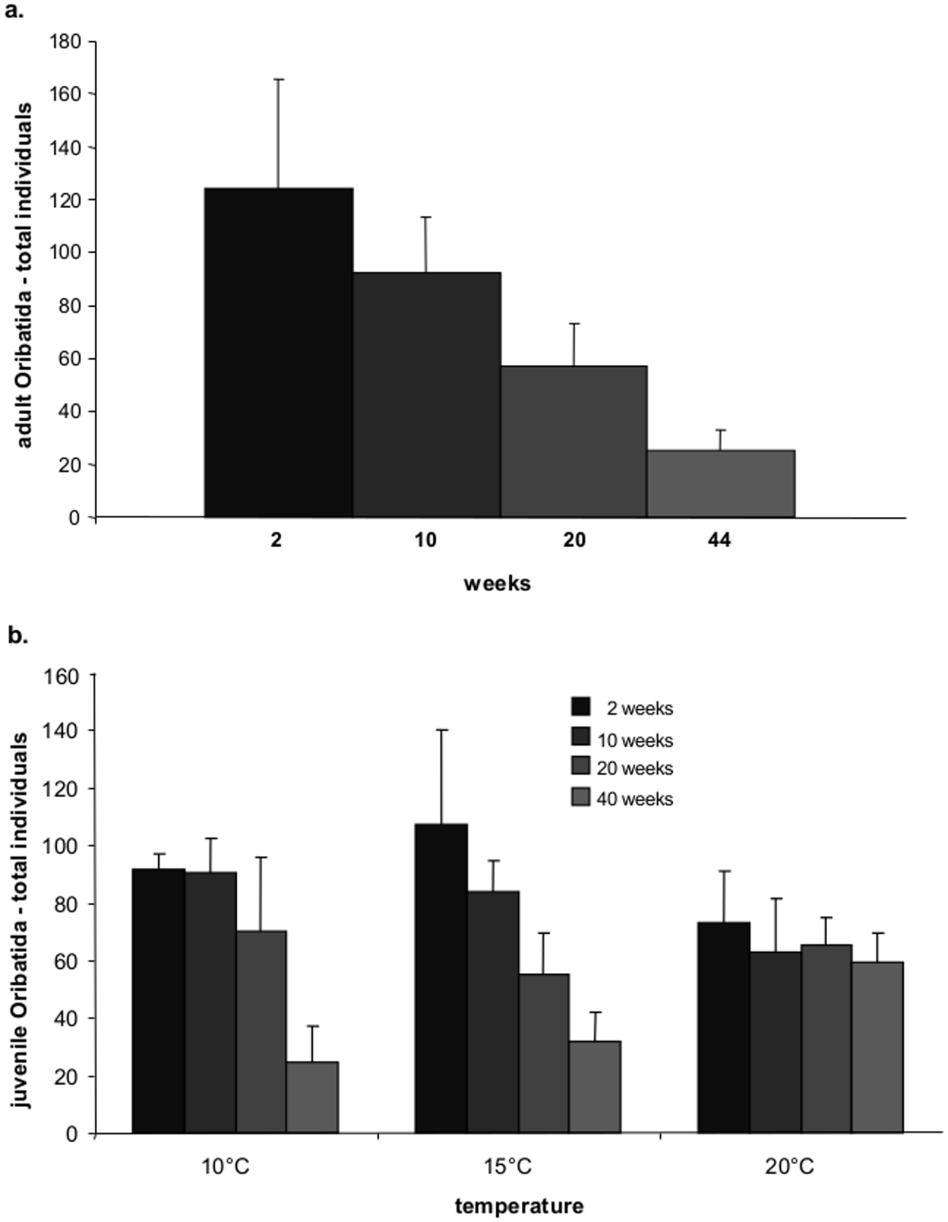
Abundance of oribatid mites. Changes in the abundance of (a) adult oribatid mites (pooled for temperature and resource treatments, see text for details), and (b) juvenile oribatid mites at 10, 15 and 20°C (pooled for resource treatments).

**Figure 3:**
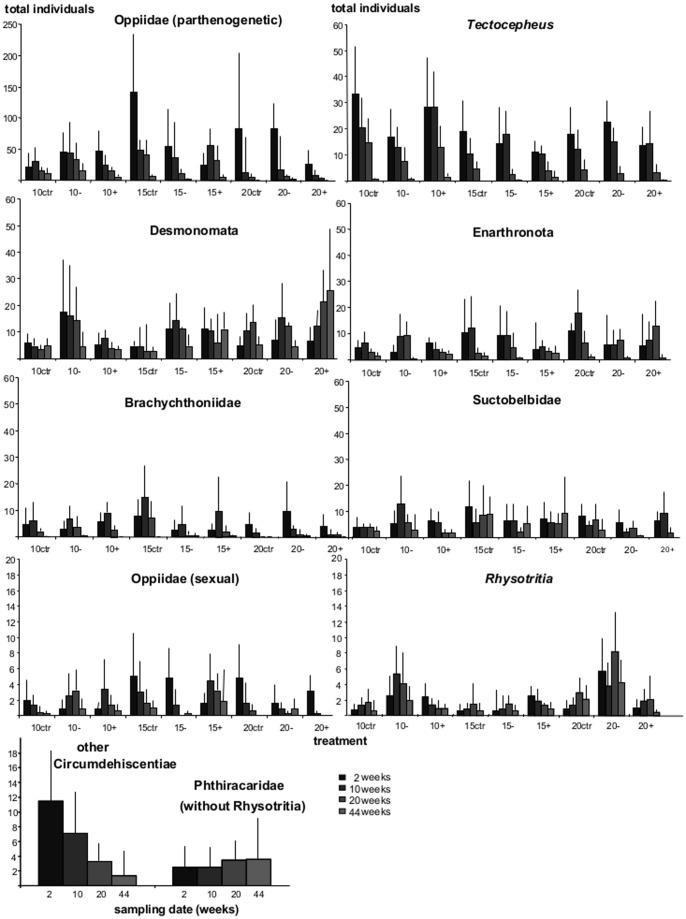
Abundance of oribatid mite subgroups. Changes in density of ten oribatid mite subgroups (sorted by abundances; see Materials and Methods) as affected by resource manipulation (control, reduced litter quality, glucose addition) and temperature (10, 15, 20°C). Data for Circumdehiscentiae and Phthiracaridae were pooled for temperature and resource treatments (see text for details). Note different scales. “ctr” =  control, “−” =  reduced litter quality, “+” =  glucose addition.

Total abundance of adult oribatid mites decreased significantly with time (RM-ANOVA, F_3,132_ = 93.3, P<0.0001), mainly between week 10 and 20 (RM-ANOVA, F_1,44_ = 37.8, P<0.0001) and 20 and 44 (RM-ANOVA, F_1,44_ = 102.7, P<0.0001; Fig. 2a). It was neither significantly affected by treatment (RM-ANOVA, F_2,36_<0.1, P = 0.985) nor by temperature (RM-ANOVA, F_2,36_ = 0.6, P = 0.542). Parthenogenetic Oppiidae, *Tectocepheus* spp. and Desmonomata were most abundant while Brachychthoniidae, *Rhysotritia*, sexual Oppiidae and Phthiracaridae were least abundant (Table S1, Fig. 3). Total numbers of juvenile oribatid mites also significantly decreased with time at 10 and 15°C but remained constant at 20°C (RM-ANOVA, F_3,108_ = 30.5, P<0.0001 for time and F_6,108_ = 4.7; P = 0.0003 for the interaction between time and treatment; Fig. 2b).

Since the interaction between time, taxa, treatment and temperature was significant (double repeated ANOVA with the repeated factors taxa and time, F_108,972_ = 1.4, P = 0.009), the development of all ten oribatid mite subgroups was analyzed separately. Subgroups of oribatid mites changed significantly with time but responded differently to treatment and temperature (Table 1, Fig. 3). In sexuals, Circumdehiscentiae and Phthiracaridae similarly responded to the treatments at each of the temperatures; while total abundance of Circumdehiscentiae significantly decreased with time, total numbers of Phthiracaridae slightly increased. Sexual Oppiidae responded significantly to time, temperature and treatment. Abundances generally declined in the control treatment while they increased until week 10 and declined thereafter in the glucose treatment at 10 and 15 but not at 20°C. The response in the reduced litter quality treatment varied with temperature; while abundances increased until week 20 and declined thereafter at 10°C, they continuously declined at 15°C, but recovered to week 44 at 20°C.

**Table 1:** F- and P-values on the effect of time (2, 10, 20 and 44 weeks), temperature (temp; 10, 15, 20°C) and treatment (tr; control, reduced litter quality and glucose addition) of the density of ten pooled oribatid mite groups analyzed by repeated measures analysis of variance.

		Brachychthoniidae		Enarthronota		*Rhysotritia*		Desmonomata	
	df	F	P	F	P	F	P	F	P
*between subject effects*									
temp	2,36	3.72	**0.0340**	0.48	0.6241	4.77	**0.0145**	1.92	0.1612
tr	2,36	0.42	0.0551	0.33	0.7239	8.29	**0.0011**	2.35	0.1100
temp x tr	4,36	3.92	**0.0097**	0.19	0.9430	0.72	0.5850	1.26	0.3051
*within subject effects*									
time	3,108	35.08	**<0.0001**	33.94	**<0.0001**	3.70	**0.0139**	3.84	**0.0118**
time x temp	6,108	2.95	**0.0105**	2.01	0.0711	1.34	0.2439	3.79	**0.0018**
time x tr	6,108	0.48	0.8194	3.02	**0.0091**	1.45	0.2014	2.90	**0.0116**
time x temp x tr	12,108	1.36	0.1968	1.11	0.3569	0.84	0.6063	1.20	0.2953

		*Tectocepheus*		Suctobelbidae		Oppiidae (parth)		Oppiidae (sex)	
	df	F	P	F	P	F	P	F	P
*between subject effects*									
temp	2,36	6.51	**0.0039**	1.20	0.3121	11.57	**0.0001**	2.64	0.0850
tr	2,36	0.53	0.5931	0.79	0.4625	1.37	0.2682	0.21	0.8147
temp x tr	4,36	0.74	0.5686	0.50	0.7358	3.12	0.0265	1.15	0.3498
*within subject effects*									
time	3,108	194.55	**<0.0001**	10.75	**<0.0001**	76.48	**<0.0001**	14.02	**<0.0001**
time x temp	6,108	2.93	**0.0109**	3.36	**0.0045**	7.23	**<0.0001**	3.51	**0.0048**
time x tr	6,108	1.58	0.1601	1.45	0.2032	0.38	0.8587	1.01	0.4213
time x temp x tr	12,108	0.67	0.7738	1.17	0.3162	1.39	0.1798	2.43	0.0106

		Circumdehiscentiae		Phthiracaridae	
	df	F	P	F	P
*between subject effects*					
temp	2,36	1.45	0.2489	2.36	0.1088
tr	2,36	1.74	0.1899	2.86	0.0704
temp x tr	4,36	0.72	0.5846	0.55	0.6990
*within subject effects*					
time	3,108	91.06	**<0.0001**	2.71	**0.0489**
time x temp	6,108	1.22	0.3028	1.56	0.1653
time x tr	6,108	1.05	0.4001	0.51	0.7993
time x temp x tr	12,108	1.54	0.1206	1.16	0.3219

Significant effects are given in bold.

In parthenogenetic species the total number of individuals declined with time. Brachychthoniidae, *Tectocepheus*, Suctobelbidae and parthenogenetic Oppiidae were strongly affected at higher temperatures as indicated by fast decline in total individuals; overall, while densities of Brachychthoniidae, Suctobelbidae and parthenogenetic Oppiidae were at a maximum at 15°C, the density of *Tectocepheus* was at a maximum at 10°C (Fig. 3). In contrast, the densities of Enarthronota, *Rhysotritia* and Desmonomata were highest at 20°C, with Desmonomata continuously increasing in the glucose treatment. Enarthronota responded differently to time and treatment. While densities increased from week 2 to 10 but decreased thereafter in control treatments, the decrease was retarded (after week 20) in the reduced litter quality and glucose treatments.

**Figure 4:**
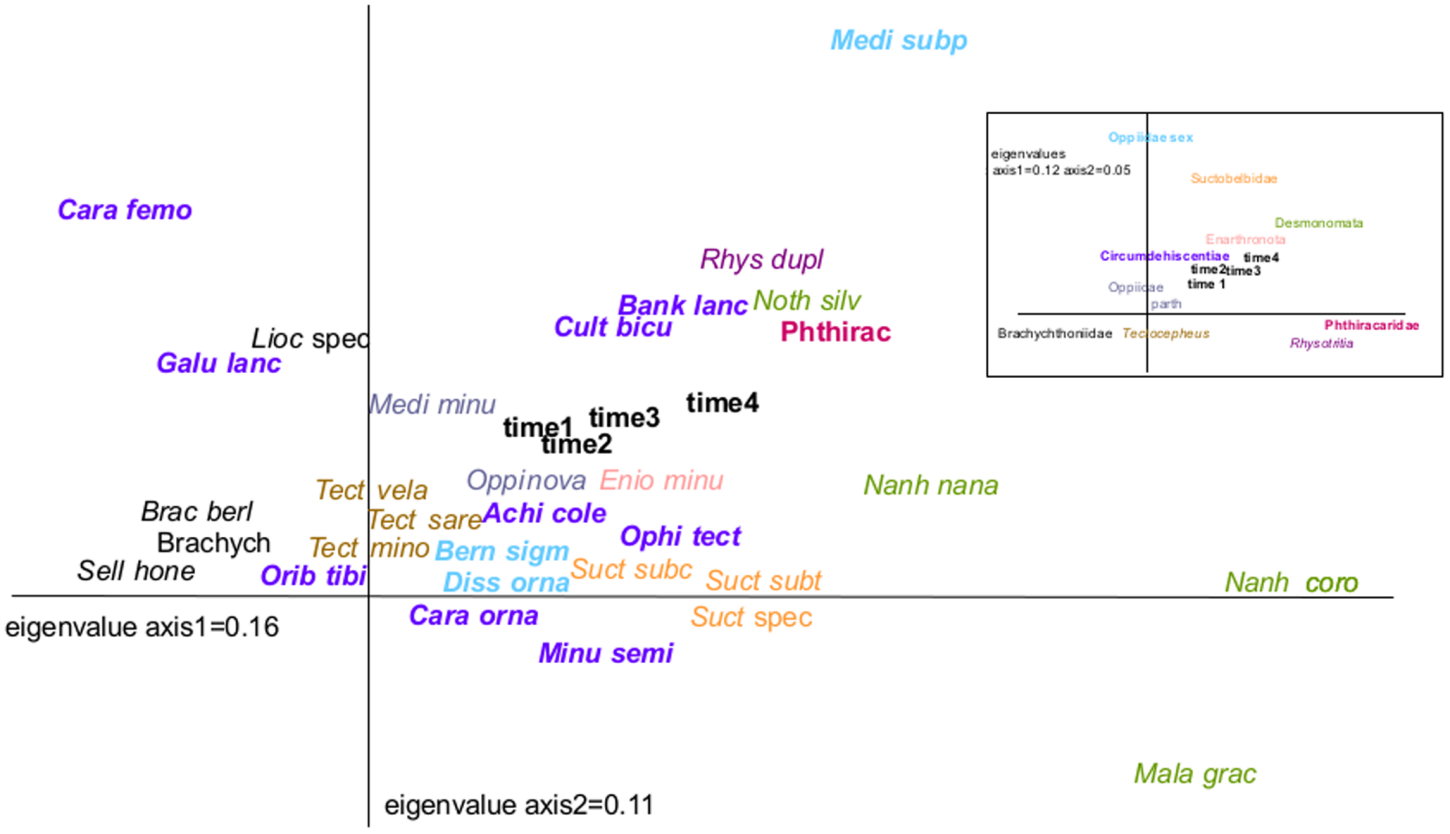
Response of oribatid mite species. Detrended correspondence analysis of oribatid mite species and subgroups (given in different colours; see small graph in figure) with sampling time included as supplementary variable not affecting the ordination (time 1 = 2 weeks, time 2 = 10 weeks, time 3 = 20 weeks, time 4 = 44 weeks). Bold names indicate sexual reproduction. *Ache cole*  =  *Achipteria coleoptrata*, *Bank lanc*  =  *Banksinoma lanceata, Bern sigm*  =  *Berniella sigma*, *Brac berl*  =  *Brachychthonius berlesei*, Brachych  =  Brachychthoniidae, *Cara femo*  =  *Carabodes femoralis*, *Cara orna*  =  *Carabodes ornatus*, *Cult bicu*  =  *Cultroribula bicultrata*, *Diss orna*  =  *Disshorina ornata, Enio minu*  =  *Eniochthonius minutissimus, Galu lanc*  =  *Galumna lanceata*, *Lioc* spec  =  *Liochthonius* sp., *Mala grac*  =  *Malaconothrus gracilis, Medi subp*  =  *Medioppia subpectinata, Medi minu*  =  *Microppia minus, Minu semi*  =  *Minunthozetes semirufus*, *Nanh coro*  =  *Nanhermannia coronata, Nanh nana*  =  *Nanhermannia nana, Noth silv*  =  *Nothrus silvestris*, *Ophi tect*  =  *Ophidiotrichus tectus, Oppi nova*  =  *Oppiella nova, Orib tibi*  =  *Oribatula tibialis*, Phthirac  =  Phthiracaridae, *Rhys dupli*  =  *Rhysotritia duplicata*, *Sell hone*  =  *Sellnickochthonius honestus*, *Suct* spec  =  *Suctobelbella* sp., *Suct subc*  =  *Suctobelbella subcornigera, Suct subt*  =  *Suctobelbella subtrigona, Tect mino*  =  *Tectocepheus minor*.

Discriminant function analysis (DFA) indicated significant changes in community structure between week 10 and 20 (F_4,29_ = 3.6, P = 0.017) and week 20 and 44 (F_4,29_ = 5.7, P = 0.002). Ordination by detrended correspondence analysis (DCA) further showed a shift in oribatid mite subgroups; while parthenogenetic Brachychthoniidae (e.g., *Brachychthonius berlesei, Sellnickochthonius honestus*) and the genus *Tectocepheus* were associated with early sampling dates (Fig. 4), parthenogenetic Desmonomata (e.g., *Malaconothrus gracilis, Nanhermannia nana, N. coronata*) and the parthenogenetic genus *Rhysotritia* were associated with later samplings dates. Sexual Phthiracaridae and *M. subpectinata* also occurred late while sexual Circumdehiscentiae (e.g., *Minunthozetes semirufus, Achipteria coleoptrata, Ophidiotrichus tectus*) were associated with early to mid sampling dates.

### Numbers of eggs

**Figure 5:**
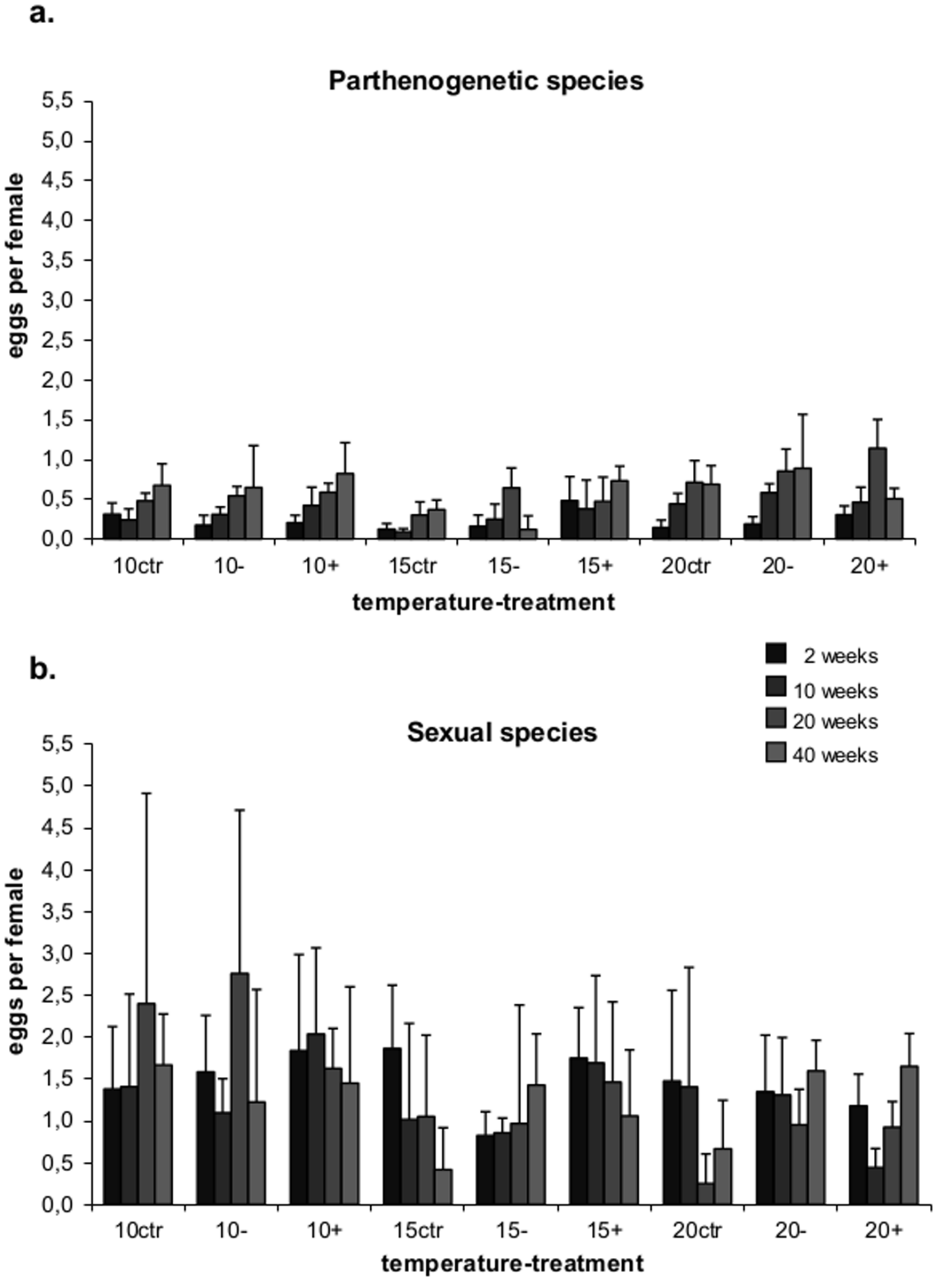
Numbers off eggs. Changes with time in the average number of eggs per female (including females without eggs) in control, reduced litter quality and glucose addition treatments at 10, 15 and 20°C in (a) parthenogenetic and (b) sexual oribatid mites.

Sexual individuals on average carried significantly more eggs per gravid female (2.21) than parthenogenetic species (1.23; RM-GLM, F_1,88_ = 183.8, P<0.0001). This held true when including females without eggs (respective values of 1.36 and 0.46; RM-GLM, F_1,64_ = 168.5, P<0.0001). While the average number of eggs per female (including those without eggs) in parthenogenetic species slightly increased with time, it remained constant in sexual species (RM-GLM, F_3,192_ = 4.0, P = 0.008 for the interaction between time and reproductive mode). However, the number of eggs changed significantly with time, temperature and treatment (RM-GLM, F_12,108_ = 2.5, P = 0.006 and F_9,45_ = 2.7, P = 0.013 for the interaction between time, temperature and treatment in parthenogenetic and sexual species, respectively). In general, in parthenogenetic species the number of eggs increased with time except in the glucose treatment at 15°C (RM-ANOVA, F_3,108_ = 37.8, P<0.0001; Fig. 5a); the increase was faster and resulted in higher egg numbers at 20°C (RM-ANOVA, F_6,108_ = 4.2, P = 0.0007 for the interaction between time and temperature; Fig. 5a). In contrast to parthenogenetic species, the number of eggs per female in sexual species (including those without eggs) was higher at lower temperatures with the maximum being at 10°C (RM-GLM, F_2,15_ = 26.5, P<0.0001; Fig. 5b). Generally, the number of eggs either remained constant (e.g., reduced litter quality treatment at 20°C) or decreased with time (e.g., control at 15°C and 20°C; RM-GLM, F_2,15_ = 45, P = 0.03 for treatment, F_3,13_ = 3.2, P = 0.052 for the interaction between temperature and treatment; Fig. 5b).

## Discussion

### Density and community structure of oribatid mites

In general, almost all parthenogenetic species comprised exclusively females, whereas sexuals had at least 10% males. However, except in few species (e.g., *Achipteria coleoptrata* and *Disshorina ornata*), sexual taxa also comprised predominantly females (e.g., 90% in *Cultroribula bicultrata* and 72% in *Oribatula tibialis*). Low percentages of females in *Carabodes ornatus* and *Oribatella quadricornuta* may be a sampling artifact due to low numbers of individuals analysed.

In the parthenogenetic species *Oppiella nova* and *Tectocepheus minor* 2% and 0.7% males were present, respectively. These rare males probably represent non-functional “spanandric” males [45]. As in other parthenogenetic animal species, spanandric males occur in a number of parthenogenetic oribatid mite species within the parthenogenetic species clusters of Enarthronota, Desmonomata and Circumdehiscentiae, e.g., in *Hypochthonius rufulus*, *Nothrus silvestris*, *Platynothrus peltifer, Trhypochthonius tectorum* and *Tectocepheus velatus* [46], but see [47]. In *O. nova* males have been reported by Fujikawa [48] with their density varying with season probably due to changes in the availability of food resources. Of the 35 males of *O. nova* found in the present study 24 occurred in the treatment with reduced litter quality, seven in the untreated control and only four in the high quality litter treatment. This supports the findings of Fujikawa [48] that the occurrence of males in *O. nova* varies with resource availability and is favoured by resource shortage similar to species reproducing by cyclical parthenogenesis, such as cladocerans [49], [50] and aphids [51], [52].

We expected oribatid mite density to change parallel to resource quality, i.e. to decline in the untreated and reduced litter quality treatment but to increase in the glucose addition treatment in particular at higher temperatures. In contrast to these expectations, the density of oribatid mites uniformly declined in each treatment at each of the temperatures indicating that the addition of glucose did not increase the availability of food resources of oribatid mites. Furthermore, sexual and parthenogenetic species responded similarly, i.e. parthenogenetic species did not outnumber sexuals in the glucose treatment and the number of sexuals declined parallel to the number of parthenogenetic species with increasing resource shortage.

Decline in density due to low rate of reproduction caused by difficulties in finding mating partners is unlikely. The process of finding mating partners in oribatid mites is not well understood, but studies indicate that males deposit spermatophores on the forest floor and females pick them up with their ovipositor without having contact to the male [21], [53]. Small-scale mobility of oribatid mites is high and high frequency of sexual species at low density as shown by Maraun et al. [19] indicates that finding spermatophores is unproblematic. Furthermore, removing a soil core of 5 cm from our 20 cm diameter microcosms unlikely affected oribatid mite communities as Schneider et al. [54] showed that oribatid mite communities are even little affected by being confined into soil cores of a diameter of 5 cm.

The impact of different temperatures on the community fluctuations was not as strong as expected. High temperature, such as 15°C and especially 20°C, may detrimentally affect oribatid mites of temperate forests. However, density and diversity of oribatid mites declined uniformly irrespective of temperature suggesting that mortality was not related to temperature in the temperature range studied [35], [55], [56].

Our expectations were based on the assumption that higher genetic diversity due to outcrossing and recombination allows sexuals to exploit a wider range of resources [2], [10], [32]. Results of previous studies suggested that parthenogenetic species indeed suffer more from resource shortage than sexual species [19], [25], for example at higher altitudes in tropical montane rainforests [20]. Resource availability may also explain the high abundance of parthenogenetic oribatid mite species in soil (with abundant resources) and the prevalence of sexual taxa on the bark of trees (with resources being in short supply) [20], [24], [25], [37]. The fact that in our experiment the density of virtually all taxa declined in each of the treatments indicates that resources uniformly declined and glucose addition did not replenish carbon resource pools used as food substrate by oribatid mites. Indeed, there is increasing evidence that decomposer animals, including oribatid mite species, heavily rely on root derived rather than leaf litter based resources [14], [15].

Despite the rather uniform response of most species in the present study, the community structure of oribatid mites shifted over time: small parthenogenetic species, such as Brachychthoniidae and the genus *Tectocepheus*, dominated early in the experiment while the larger parthenogenetic Desmonomata and *Rhysotritia* became more dominant later. Presumably, this reflects different trophic niches of these oribatid mite groups with species of the first group predominantly exploiting easily available food resources whereas species of the latter also exploit recalcitrant litter resources which is confirmed by recent findings based on stable isotope analysis [15], [57].

### Numbers of eggs

In agreement with previous studies [25], the number of eggs per female in sexual taxa on average exceeded that in parthenogenetic taxa and this was true throughout the experiment. In sexual species most eggs were produced at low temperatures, indicating higher resource availability at low temperature. In contrast, in parthenogenetic species the average number of eggs per female significantly increased during the experiment and this was independent of the addition of glucose. In general, the number of eggs in parthenogenetic species was at a maximum at high temperature indicating increased reproduction. The increase in egg production during the experiment further indicates that parthenogenetic taxa adjusted the investment in reproduction to alterations in the availability of resources which is consistent with earlier results [25]. The fact that the density of parthenogenetic species declined parallel to that of sexual species despite the number of eggs per female in the former increased indicates that offspring of parthenogenetic species suffered from higher death rates than those of sexuals. However, although sexual taxa produced more eggs than parthenogenetic species, the rates of egg deposition and therefore population growth of sexual vs. parthenogenetic species remains unknown.

## Conclusions

Manipulation of resource availability by the addition of glucose neither affected the performance of sexual nor that of parthenogenetic oribatid mites. Rather, both sexually and parthenogenetically reproducing species uniformly suffered during laboratory incubation suggesting that most oribatid mite species rely on root associated resources such as mycorrhiza rather than on detritus and associated saprotrophic fungi. Contrasting the view that parthenogenetic species reproduce faster than sexual species, parthenogenetic oribatid mites produced fewer eggs than sexual species with the number of eggs increasing later in the experiment and at higher temperature, i.e. when resources were depleted. The ability to adjust the investment in reproduction by changing egg production supports the hypothesis that resource availability differentially affects sexually and pathenogenetically reproducing species and therefore the view that the mode of reproduction is intricately bound to the availability of resources.

## Supporting Information

Table S1 **Sex ratio and numbers of eggs of oribatid mite species.**
(PDF)
