# Supplementary material for: Resource Availability as Driving Factor of the Reproductive Mode in Soil Microarthropods (Acari, Oribatida)
Source: PLoS One. 2014 Aug 6;9(8):e104243. doi: 10.1371/journal.pone.0104243 (PMC4123916; doi:10.1371/journal.pone.0104243)
Supplement: Table S1 — Sex ratio and numbers of eggs of oribatid mite species. (PDF) [file pone.0104243.s001.pdf]

Supplementary Material Table S1. Sex ratio and numbers of eggs of oribatid mite species.

	Species	female	male	sex unknown	total	total sexed	%female°	eggs[+]	egg/female[#]	egg/population[§]
**Enarthronota**										
Brachychthoniidae	*Brachychthonius berlesei* Willmann, 1936	54	0	298	352	54	100	49	1,00	0,91
	Brachychthoniidae Thor, 1934	7	0	140	147	7	100	5	1,00	0,71
	Liochthonius sp. v. d. Hammen, 1959	1	0	13	14	1	100	1	1,00	1,00
	Sellnickochthonius honestus (Moritz, 1976)	8	0	85	93	8	100	8	1,00	1,00
Eniochthoniidae	*Eniochthonius minutissimus* (Berlese, 1903)	382	0	600	982	382	100	210	1,00	0,55
Hypochthoniidae	*Hypochthonius rufulus* C.L. Koch, 1835[$]	4	0	5	9	4	100	1	1,00	0,25
**Mixonomata**										
Euphthiracaridea	*Rhysotritia duplicata* (Grandjean, 1953)	367	0	10	377	367	100	420	1,71	1,14
Phthiracaridae	Phthiracaridae Perty, 1841	244	0	291	535	244	100	429	1,81	1,76
	Steganacarus magnus (Nicolet, 1955)[$]	2		1	3	2	100	5	5,00	2,50
**Desmonomata**										
Malaconothridae	*Malaconothrus gracilis* v.d. Hammen, 1952	242	0	31	273	242	100	50	1,02	0,21
Nanhermanniidae	*Nanhermannia coronata* Berlese, 1913	41	0	0	41	41	100	30	1,36	0,73
	Nanhermannia nana (Nicolet, 1855)	548	0	0	548	548	100	390	1,30	0,71
Nothridae	*Nothrus silvestris* Nicolet, 1855	737	0	0	737	737	100	1169	2,08	1,59
**Circumdehiscentiae**										
Achipteriidae	*Achipteria coleoptrata* (Linnaeus, 1758)	171	127	3	313	310	55,16	311	2,27	1,82
Astegistidae	*Cultroribula bicultrata* (Berlese, 1905)	38	4	0	42	42	90,48	11	2,20	0,29
Carabodidae	*Carabodes areolatus* Berlese, 1916[$]	0	2	2	4	2	0,00	0		
	Carabodes femoralis (Nicolet, 1855)	4	5	6	14	8	50,00	7	3,50	1,75
	Carabodes ornatus Storkan, 1925	2	5	4	11	7	28,57	1	1,00	0,50
Galumnidae	*Galumna lanceata* Oudemans, 1900	10	10	4	24	20	50,00	7	2,33	0,70
Oppiidae	*Berniniella sigma* (Strenzke, 1951)	49	57	0	105	105	46,67	2	1,00	0,04
	Dissorhina ornata (Oudemans, 1900)	88	75	0	169	169	52,07	98	1,61	1,11
	Medioppia subpectinata (Oudemans, 1900)	13	10	0	23	23	56,52	16	2,00	1,23
	Microppia minus (Paoli, 1908)	3209	0	22	3231	3209	100	22	1,00	0,01
	Oppiella nova (Oudemans, 1902)	1812	35	6	1855	1849	98,00	414	1,19	0,23
Oribatellidae	*Ophidiotrichus tectus* (Michael, 1884)	11	11	0	22	22	50,00	8	2,67	0,73
	Oribatella quadricornuta Michael, 1880[$]	1	1	0	3	3	33,33	4	4,00	4,00
Oribatulidae	*Oribatula tibialis* (Nicolet, 1855)	244	93	16	353	337	72,40	434	3,50	1,78
Phenopelopidae	*Eupelops plicatus* (C.L. Koch, 1836)[$]	3	0	1	4	3	100	0		0,00
Punctoribatidae	*Minunthozetes semirufus* (Koch, 1841)	53	39	3	95	92	57,61	23	2,09	0,43
Suctobelbidae	Suctobelbidae Jacot, 1938	3	0	180	183	3	100	3	1,00	1,00
	Suctobelbella subcornigera (Forsslund, 1941)	17	0	595	612	17	100	17	1,00	1,00
	Suctobelbella subtrigona (Oudemans, 1900)	7	0	156	163	7	100	7	1,00	1,00
Tectocepheidae	*Tectocepheus minor* (Berlese, 1903)	289	2	0	291	291	99,31	115	1,15	0,40
	Tectocepheus sarekensis Trägardh, 1910	451	0	0	451	451	100	52	1,24	0,12
	Tectocepheus velatus (Michael, 1880)	1146	0	1	1147	1146	100	103	1,11	0,09
Thyrisomidae	*Banksinoma lanceata (Michael, 1888)*	86	76	27	189	162	53,09	45	1,50	0,52

[$] species are excluded from all statistical analyses

° from sex-determinable specimens

[+] total number

[#] only females with eggs

[§] total number of females including those without eggs
